# The Relationship between Mustard Import and COVID-19 Deaths: A Workflow with Cross-Country Text Mining

**DOI:** 10.3390/healthcare10102071

**Published:** 2022-10-18

**Authors:** Ge Zhan, Fuming Yang, Liangbo Zhang, Hanfeng Wang

**Affiliations:** 1 AI Data Analytics Lab, Beijing Normal University-Hong Kong Baptist University (BNU-HKBU United International College), Zhuhai 519087, China; 2 Division of Science & Technology, Beijing Normal University-Hong Kong Baptist University (BNU-HKBU United International College), Zhuhai 519087, China; 3 School of Economics and Management, Harbin Institute of Technology Shenzhen, Shenzhen 518000, China

**Keywords:** text-mining, knowledge graph, COVID-19, natural foods

## Abstract

We developed a workflow for the search and screening of natural products by drawing from worldwide experiences shared by online platform users, illustrated how to cope with COVID-19 with a text-mining approach, and statistically tested the natural product identified. We built a knowledge base, which consists of three ontologies pertaining to 7653 narratives. Mustard emerged from texting mining and knowledge engineering as an important candidate relating to COVID-19 outcomes. The findings indicate that, after controlling for the containment index, the net import of mustard is related with reduced total and new deaths of COVID-19 for the non-vaccination time period, with considerable effect size (>0.2).

## 1. Introduction

Many developing economies have inadequate doses to vaccinate their populations [1]. Countries with high vaccination rates are experiencing a new round of the outbreak, largely caused by new COVID-19 variants such as Delta and Lambda. Recent research indicates the potential for COVID-19 variants to escape from neutralizing humoral immunity [2,3], and the effectiveness of vaccination has been found to be lower among people with the Delta variant than those with the Alpha variant [4]. There is an urgent need for non-vaccination interventions against this disease [1].

Drug-repurposing opportunities identified in previous studies need to be evaluated over time, and clinical trials are still in progress [5,6]. While there are a huge amount of COVID-19 research works published on drug discovery, few studies have suggested exploiting potential natural products [7]. Previous works on natural product identification have proposed approaches mining genome and metabolomics data [8,9]. However, such methods are limited by the scope of their search and are usually time-consuming. A more efficient or novel way of gaining insights from data in healthcare research is mining and analyzing online text with natural language processing (NLP) or text analytics [10]. For example, researchers of recent healthcare studies have learned public opinion and sentiment on topics such as COVID-19 vaccine boosters [11] and intimate partner violence [12] by mining textual data from social media.

In this study, we developed a new workflow for the search and screening of natural products from 7653 narratives that can be potentially used to cope with COVID-19. To illustrate this approach, we explored online narrative texts collected from a large tourism platform, identified a potential natural food with a knowledge graph approach, and tested it statistically. We hypothesize that natural food consumption is related to COVID-19 outcomes. We then test the data on COVID-19 deaths and cases drawn from John Hopkins University (JHU) database.

The number of medicine discovery projects on the basis of big data and data mining has been growing in recent years [13,14]. However, most studies so far built their databases by collecting published COVID-19 studies [15,16]. We compiled a dataset by visiting a large Chinese tourism website wherein a large population of users upload and share a large amount of travel writings that state their experiences with various overseas destinations. The use of online travel writing helps us to efficiently gain insights into the consumption of some “special” local food or consumption culture for each destination [17,18]. The content downloaded includes 7653 travel writings, as well as the upload date and destination. The data were grouped and combined according to destination, and each document consists of all the travel writings of a particular country. In total, we compiled a database representing 209 countries, covering all major regions and cultures in the world. The dataset reflects local condition within a time period before the pandemic, so this type of data was considered more appropriate than Wikipedia, wherein we cannot efficiently learn in-depth information on natural products in each location and can not specify a particular time period.

## 2. Methods

### 2.1. A Workflow with NPN Approach

**Figure 1:**
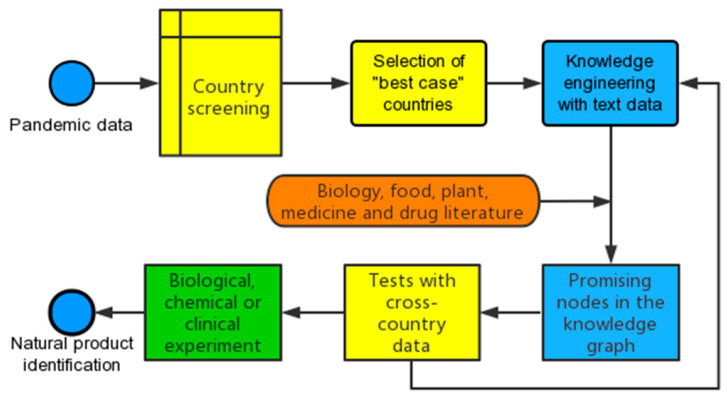
A workflow for exploring natural products narratives (NPN). Yellow boxes represent the use of cross-country data for the screening and selection of “best case” countries, e.g. those with lower confirmed cases and deaths, and for statistical testing. Blue boxes show a knowledge engineering process that transforms text into structured data and then visualizes promising nodes (natural products) in the knowledge graph. The orange box indicates domain knowledge in the knowledge engineering process. The green box shows experiments needed before confirming the identification of natural products.

As the workflow (Figure 1) is used to isolate the best cases of countries from natural products narratives (NPN), which may provide us hints on natural products, we deleted countries with highly restrict responses to COVID-19. As successful government intervention such as closing schools and banning gatherings are highly effective at controlling community transmission during COVID-19 [19,20,21], countries with a lower containment and health index are considered more promising for the detection of natural solutions. We used the Containment and Health Index, which was developed by the Oxford Coronavirus Government Response Tracker (OxCGRT) project, and a higher value of the index indicates a stricter response to the pandemic (100 for strictest response). The index is based on thirteen policy-response indicators including school closures, workplace closures, travel bans, testing policy, contact tracing, face coverings, and vaccine policy. If countries with low containment observe fewer COVID-19 deaths or cases, there might be some unknown causes. Countries that did not achieve 50% in the COVID Data Transparency Index (totalanalysis.com/Covid19/TAIndex, accessed on 5 August 2021) were deleted in data mining, as the numbers of deaths and confirmed cases in these countries might not be accurate.

We end up with a final list of countries with both a low containment index and low total COVID-19 confirmed cases (Table 1). Although African countries lack effective containment and health facilities, many of them have surprisingly low level of total cases per million (<1000), e.g. Burundi, Burkina Faso, Congo Dem. Rep., Madagascar, Niger, Senegal, Somalia, Sudan, and Tanzania. S1 figures (supporting information [22]) show that most of the selected countries observe less COVID-19 deaths than the world average.

### 2.2. Data Analysis

Knowledge engineering as a sub-field of artificial intelligence involves transferring human knowledge into a database and representing expert knowledge and reasoning [23]. We adopt knowledge engineering logic to develop a system to sort text data, isolate knowledge, and compile a domain-specific knowledge base. The knowledge base consists of three ontologies: food, drink, and smell. While natural food and drink might serve as medicine, substances in the air may influence the process by which a virus spreads from infected people to others. NLTK's (Natural Language Toolkit) tokenizers were used to convert narrative text into structured data. All files were then merged on the basis of these ontologies. This approach enabled us to connect concepts among sub-datasets and identify natural products spanning several countries, which has been a common limitation of previous knowledge mining studies [24].

## 3. Results

### 3.1. Corpus Development and Knowledge Engineering

We developed a corpus of Chinese words related to eat, drink, food, smell, dish, cooking, taste, etc. The corpus was employed to screen out the sentences that do not consist of food, drink, and smell information. These invalid sentences were dropped from the knowledge base. Then, seven research assistants (college students) were recruited and trained. They manually annotated each valid sentence with tags indicating the actual product name used among local people and classified the tags under each ontology. The annotated data were checked and verified until inter-rater reliability was over 90%.

**Figure 2:**
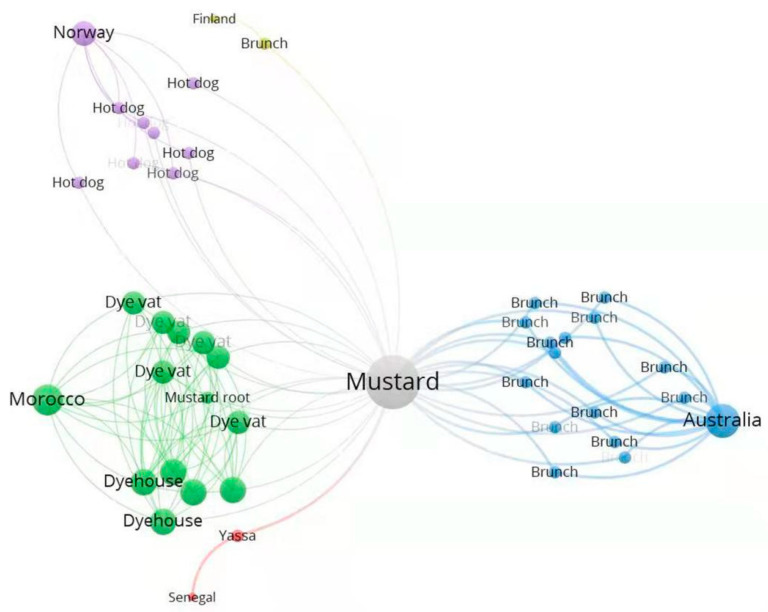
Knowledge graph of nodes connected to mustard. The graph was made by VOSviewer [25,26]. Circle size reflects the number of links. Nodes from the same country are clustered with the same color.

The purpose of the knowledge engineering was to identify similarities from the sample countries in terms of food-consumption culture. The more documents connected to a node, the more important the factor should be. A notable challenge with the text-mining approach was isolating good candidates from the large amount of nodes. We removed those nodes that are commonly consumed globally. A good candidate of potential factors identified from the data is mustard, which emerges as an important node linking multiple documents/countries from different angles (Figure 2). Mustard has been mentioned frequently in narratives about Moroccan food and its dye industry. It has also been noted in other narratives as common ingredients in Senegal’s Yassa (local dish), hot dog in Norway, and brunch in Finland and Australia.

Mustard consumption is measured by net import, which is defined as imports minus exports of mustard. Net import data (product category: 210,330–mustard flour and meal and prepared mustard, by country) were provided by World Integrated Trade Solution (WITS), which was co-developed by the World Bank and the United Nations Conference on Trade and Development (UNCTAD). Net import is a good proxy for national consumption, particularly when consumption data of a specific product category is not available [27,28].

We tested the relationship between net import of mustard and COVID-19 deaths worldwide. If the nutrition and consumption of this food are related to the prevention, or cure, of this disease, then the net import of this mustard should negatively influence the number of COVID-19 deaths. The first COVID-19 vaccination took place in the mid of December 2020. To test the pure effect of mustard, we collected worldwide COVID-19 data from 1 March to 10 December 2020, a time period before vaccination.

### 3.2. Hypothesis Testing

The fruits or vegetables mentioned in the narratives pertaining to countries with low levels of COVID deaths and containment index include cassava, pepper, cabbage, papaya, banana, orange, avocado, olive, cocoa, coconut, pineapple, mango, apple, watermelon, almonds, tomato, cucumber, shallot, fennel, pepper, potato, cherry, beans, corn, ginger, cinnamon, calyx pear, chamomile, sisal, eggplant, radish, red pomelo, tricholoma matsutake, jujube, jackfruit, avocado, litchi, plum, sugarcane, polo, baobab fruit, sakya, papaya, kiwifruit, and cactus fruit. We checked and compared with the WITS food list and tested those foods for which we could find valid data from the WITS database. Both import and export data in 2018 were downloaded. The food category consisting of multiple types of food was not considered (e.g. “080450—Fruit, edible; guavas, mangoes and mangosteens, fresh or dried”), as one can not tell which particular food might cause an effect. Some vegetables or fruits indicate non-trival but small negative associations (correlation coefficient around 0.1) on COVID deaths, such as “cucumbers/gherkins” and “coconuts”, but none of these have a correlation coefficient with COVID deaths greater than 0.2 (see Table 2).

We then analyzed other vegetables and fruits from the WITS trade database and ended up with 41 other types of foods for correlation tests. The results of grape indicate a small but considerable relationship with COVID deaths (correlation coefficient > 0.1 for total and new deaths). Again, mustard shows a much stronger relationship with COVID deaths compared with the 41 types of foods.

We used regression models in statistical tests with robust standard errors. As multiple data, i.e. daily deaths, were observed from each country, the determination of statistical significance was based on clustered-robust standard errors (clustered by country). This is more conservative, as well as more accurate usually, for hypothesis-testing [29]. We hypothesize that mustard consumption is associated with COVID-19 outcomes. The dependent variables that we investigate in the statistical test include total and new deaths of COVID-19. Correlation analyses indicate considerable associations [30] between net import of mustard and total deaths (Pearson’s *r* = −0.24, *p* < 0.05), and between net import of mustard and new deaths (Pearson’s *r* = −0.21, *p* < 0.05). The direction of the relationships is consistent with our hypothesis.

We developed two models in testing the hypothesis (Table 3). As healthy economics and demographics may assist nations with combatting the COVID-19 pandemic [31,32], we selected the following confounders: population, life expectancy, GDP per capita, cardiovascular death rate, diabetes prevalence, percent of population aged 70+, population density, and number of hospital beds (per thousand), which were drawn from Our World in Data (Global Change Data Lab). As national infrastructure may also facilitate an efficient reaction to COVID-19, we also controlled confounding factors, such as electricity and mobile subscriptions, by drawing data from World Bank.

We tested if the net import of mustard has a negative relationship with total deaths. The results indicate that Model 1 predicts a good proportion of total deaths (*r*^2^ = 0.514) and a significant and negative effect of net import on total deaths (*p* = 0.020) after controlling for all of the confounding variables above. We next tested with Model 2 if the net import of mustard could predict new deaths caused by COVID-19. The same set of variables were controlled. Again, the net import of mustard shows a significant and negative relationship with new deaths (*p* = 0.034).

### 3.3. Sensitivity Analysis for Additional COVID-19 Outcomes

COVID-19 outcomes can be alternatively measured with the number of confirmed cases. After controlling for all of the above confounding factors, the results indicate that the net import of mustard is negatively and significantly related to total (*p* = 0.046) and new cases (*p* = 0.037) (Table 4). Correlation analysis results indicate a considerable effect size for the associations between the net import of mustard and total cases (Pearson’s *r* = −0.19) and between the net import of mustard and new cases (Pearson’s *r* = −0.23).

**Figure 3:**
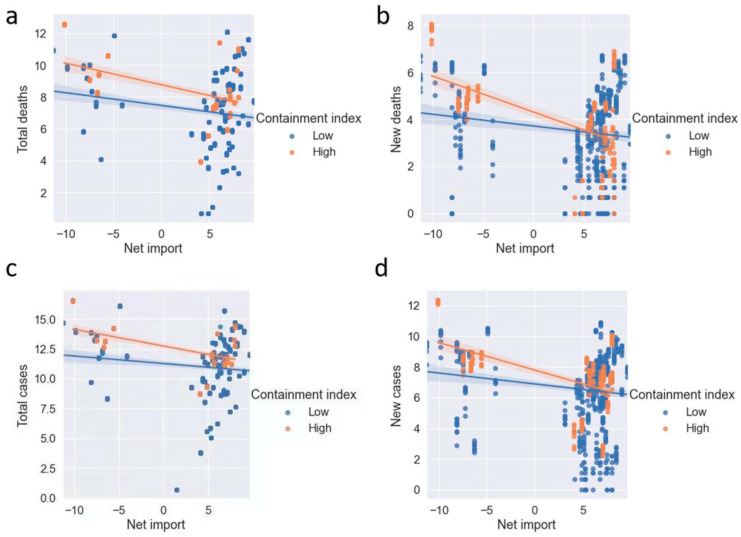
Factors correlated with the net import of mustard. (**A**,**B**) Total and new deaths caused by COVID-19 are negatively related to net import (log transformed). Brown lines represent a high containment index (>70), while blue lines represent a low containment index (≤70). (**C**,**D**) Total and new COVID-19 confirmed cases are negatively related to net import. The correlations are compared with different levels of the containment index.

As shown in previous studies, deaths and confirmed cases are strongly influenced by governmental factors such as lockdown, stringency, testing policy, the extent of contact tracing, requirements to wear face coverings, and policies around vaccine rollout [33]; we did additional tests by dividing all sample countries into two groups with a high (scored above 70) and low Containment Index (≤70). Figure 3a–d indicates that the net import of mustard consistently have negative relationships with total (new) deaths and total (new) cases for both groups of countries. The negative relationship is stronger for countries with a high containment index, with *r* ranging from −0.749 to −0.688.

## 4. Discussion

The proposed workflow can accelerate the identification of potential natural products against COVID-19. This novel method has been illustrated by drawing from experiences and writings relating to natural products on over 200 countries. Although narratives pertaining to a particular country would be contextual, a cross-country comparison of such data may bring some new and natural solutions against the disease. Since both cross-country NPN and product data can be collected from online or public sources, our approach is a general and time-efficient one, without additional inputs from metabolic engineering or gene expression.

The case of mustard provides a proof-of-concept. The net import of mustard has been found to be associated with reduced deaths, particularly when nations manage to improve the containment index to a level of above 70 (*r* becomes around −0.7). The findings are robust to the use of confirmed case data. These findings provide new explanations on why some countries, although less resourceful in terms of vaccine, crisis containment, and healthcare facilities, are not harmed seriously by the pandemic.

By examining the relationships between the net import of other foods and COVID-19 outcomes, we found several notable positive correlations (>0.2) between COVID-19 total deaths and the net import of almond (0.317), pineapple (0.292), grapes (0.277), and apples (0.238). These fruits, although not associated with a reduced number of COVID-19 deaths, might provide new hints on the factors relating to the growing number of COVID-19 deaths. Future research could address this issue by investigating the nutrition or chemical mechanisms underlying the connections between these fruits and COVID-19.

This study was limited by the scope of the destination data. Although we collected a fairly good number of travel writings on 209 nations or regions, the dataset does not cover every country in the world and does not convey a complete picture of local consumption and culture. Future studies could investigate the molecular mechanism pertaining to mustard, particularly pertaining to mustard seed, which has been used to make sauce and dye in our sample countries. Mustard has been found to be useful in the treatment of pneumonia, asthma, or cough and in relieving pain symptoms, such as headaches and neuralgia. Since fermentation with *Lactobacillus Plantarum* can enhance the anti-inflammatory activity of mustard leaves, mustard leaves fermented by this microorganism may be helpful in the treatment of inflammation [34]. The SARS-CoV-2 protein 3CLPro is essential for successful viral replication. A recent study found that a glucosinolate derivative found in mustard seeds is a potent inhibitor of SARS-CoV-2 3CLPro [35].

Future work may also try using other data-driven or algorithm-driven approaches in the identification of foods related to COVID 19. While our workflow is developed on the basis of a knowledge graph that is exploratory in nature and built on domain-specific knowledge, deep learning applications in NLP, particularly BERT models, have good potential for the efficient identification of food-related words or terms from large social network sites, such as Twitter. For example, in a recent healthcare study, researchers developed a NLP algorithm and accurately extracted sleep parameters from polysomnography text noted in electronic medical records [36].

## Figures and Tables

**Table 1:** Countries ranked by containment index.

Country Name	Containment Index	Total Cases/M	Country Name	Containment Index	Total Cases/M
Nicaragua	12.50	881	Somalia	38.69	280
Tanzania	16.37	9	Finland	44.94	4595
Burundi	17.26	58	Denmark	48.21	14,238
Yemen Rep.	18.45	70	Norway	50.60	6750
Afghanistan	23.81	1195	United Arab Emirates	52.62	17,203
Central African Rep.	24.40	1018	Netherlands	52.98	31,289
Congo Dem. Rep.	24.70	144	Singapore	52.98	9953
Sudan	26.19	416	South Africa	55.36	13,359
Mauritius	26.79	397	Sweden	57.14	25,819
Niger	27.08	66	Australia	57.14	1095
Mauritania	28.57	1873	Korea Rep.	58.63	686
Burkina Faso	28.57	140	Bahrain	59.52	51,209
New Zealand	32.14	427	Belgium	60.71	49,977
Syria	33.04	456	Lithuania	61.31	22,963
Congo Rep.	35.71	1046	Germany	61.31	13,065
Eswatini	36.90	5553	Qatar	61.90	48,246
Tajikistan	36.90	1282	Morocco	62.20	9749
Senegal	36.90	962	United Kingdom	63.10	24,265
Madagascar	36.90	626	Luxembourg	63.10	56,119
Haiti	38.10	815	Spain	63.69	35,428

Note: Total cases/m is total cases per million. Countries in the table with both a low containment index and low total COVID-19 confirmed cases are included in analysis. Countries that failed to manage data transparently (<50% in the Covid Data Transparency Index) were deleted in data mining. Group A lists countries with good data transparency (≥50%), and group B includes countries whence transparency data are not available. In both groups, the top 20 countries with the lowest containment index were listed. In group A, countries have big variance in total cases, ranging from 427 to 56,119. As the aim of data mining was to explore “best-case” countries that may provide hints of natural foods against COVID-19, we deleted countries with total confirmed cases above 10,000. Singapore and Korea Rep. have demonstrated good administrative practice in pandemic control, so both countries were removed from the sample. In group B where countries have smaller variance, we deleted those with total cases above 1000.

**Table 2:** Correlation tests between selected foods and COVID-19 outcomes.

	Potatoes	Cucumbers and Gherkins	Fruits of the Genus Capsicum or of the Genus Pimenta
	70,190	70,700	70,960
	N = 14,757	N = 12,300	N = 14,621
Total cases	0.0864	−0.0672	−0.0302
New cases	0.0946	−0.0694	−0.0465
Total deaths	0.1272	−0.106	−0.05
New deaths	0.0735	−0.0729	−0.0459
	mushrooms and truffles	coconuts	almonds
	71,230	80,110	80,212
	N = 14,191	N = 14,378	N = 14,312
Total cases	−0.0294	−0.0201	0.231
New cases	0.0636	−0.0801	0.1922
Total deaths	−0.0426	−0.0628	0.3168
New deaths	−0.0023	−0.1166	0.2081
	pineapples	oranges	citrus fruit
	80,430	80,510	80,590
	N = 13,420	N = 14,534	N = 10,325
Total cases	0.2359	−0.014	−0.0767
New cases	0.1707	−0.0022	−0.0579
Total deaths	0.2916	−0.0838	−0.0662
New deaths	0.1785	−0.074	−0.0522
	grapes	papaws	plums and sloes
	80,620	80,720	80,940
	N = 13,837	N = 8381	N = 12,994
Total cases	0.2066	0.1354	0.0083
New cases	0.227	0.1258	0.0238
Total deaths	0.2766	0.1708	0.0039
New deaths	0.2479	0.1062	0.0275
	apples		
	81,330		
	N = 11,232		
Total cases	0.1649		
New cases	0.1726		
Total deaths	0.2379		
New deaths	0.1767		

Note: WITS product codes and correlation coefficients are shown.

**Table 3:** Mustard net import and deaths caused by COVID-19.

	Total Deaths (Model 1)				New Deaths (Model 2)			
	Coef.	SE	t	*p* > t	Coef.	SE	t	*p* > t
Net import	−0.011	0.005	−2.37	0.020	−0.011	0.005	−2.17	0.034
Containment index	0.010	0.011	0.90	0.371	0.014	0.010	1.48	0.144
Log(population)	1.051	0.088	11.91	0.000	0.692	0.067	10.32	0.000
Life expectancy	−0.146	0.063	−2.33	0.023	−0.173	0.036	−4.74	0.000
Log(GDP per capita)	1.181	0.357	3.31	0.001	0.315	0.227	1.39	0.169
Cardiovasc death rate	−0.002	0.001	−1.70	0.094	−0.003	0.001	−3.69	0.000
Diabetes prevalence	−0.053	0.040	−1.33	0.189	−0.032	0.033	−0.96	0.340
Aged 70	0.097	0.051	1.90	0.062	0.089	0.032	2.78	0.007
Log (population density)	0.008	0.129	0.06	0.953	−0.115	0.093	−1.24	0.220
Hospital beds (1000)	−0.144	0.059	−2.46	0.016	−0.140	0.039	−3.55	0.001
Electricity	0.054	0.019	2.81	0.006	0.082	0.015	5.62	0.000
Mobile subscriptions	−0.023	0.008	−3.07	0.003	−0.008	0.004	−1.83	0.072

Note: Unit of net import: million USD. Total and new deaths are log transformed. The *p* values gained from a two-sided *t* test; SE clustered and adjusted for 73 countries; *p* values < 0.05 indicates statistical significance. Model 1: number of obs. = 19,530; F(12, 72) = 91.93; Prob > F = 0.000; *r*^2^ = 0.514; Root MSE = 1.981. Model 2: number of obs. = 13,412; F(12, 72) = 18.90; Prob > F = 0.000; *r*^2^ = 0.431; Root MSE = 1.480.

**Table 4:** Sensitivity analysis: confirmed cases as Covid-19 outcomes.

	Total Cases (Model 3)				New Cases (Model 4)			
	Coef.	SE	*t*	*p* > *t*	Coef.	SE	*t*	*p* > *t*
Net import	−0.010	0.005	−2.030	0.046	−0.017	0.008	−2.120	0.037
Containment index	0.012	0.010	1.210	0.228	0.007	0.018	0.370	0.710
Log(population)	0.942	0.079	11.900	0.000	0.763	0.141	5.420	0.000
Life expectancy	−0.047	0.069	−0.680	0.502	−0.159	0.072	−2.210	0.030
Log(GDP per capita)	1.502	0.348	4.310	0.000	1.383	0.417	3.310	0.001
Cardiovasc death rate	0.000	0.001	0.240	0.813	−0.001	0.001	−0.390	0.701
Diabetes prevalence	−0.020	0.041	−0.490	0.627	−0.028	0.052	−0.540	0.593
Aged 70	−0.002	0.046	−0.050	0.963	0.011	0.058	0.190	0.848
Log(population density)	0.008	0.081	0.100	0.920	−0.009	0.119	−0.080	0.940
Hospital beds (1000)	−0.074	0.054	−1.380	0.172	−0.088	0.067	−1.320	0.192
Electricity	0.011	0.019	0.600	0.553	0.039	0.020	1.990	0.050
Mobile subscriptions	−0.014	0.006	−2.130	0.036	−0.014	0.009	−1.500	0.137

Note: Unit of net import: million USD. Total and new cases are log transformed. The *p* values gained from a two-sided *t* test; SE clustered and adjusted for 74 countries; *p* values < 0.05 indicates statistical significance. Model 1: number of obs. = 20,853; F(12, 73) = 42.48; Prob > F = 0.000; *r*^2^ = 0.349; Root MSE = 2.358. Model 2: number of obs. = 18,889; F(12, 73) = 13.39; Prob > F = 0.000; *r*^2^ = 0.287; Root MSE = 2.173.

## Data Availability

Sample data and aggregated statistics for replication and academic research purposes are available from the corresponding author on reasonable request.
